# The Neural Correlates of Spatial and Object Working Memory in Elderly and Parkinson's Disease Subjects

**DOI:** 10.1155/2015/123636

**Published:** 2015-03-16

**Authors:** Silvia P. Caminiti, Chiara Siri, Lucia Guidi, Angelo Antonini, Daniela Perani

**Affiliations:** ^1^In-Vivo Human Molecular and Structural Neuroimaging Unit, Division of Neuroscienc San Raffaele Scientific Institute, Via Olgettina 58, 20132 Milan, Italy; ^2^Parkinson Institute, Istituti Clinici di Perfezionamento, Via Bignami 1, 20126 Milan, Italy; ^3^IUSS Pavia, Piazza della Vittoria 15, 27100 Pavia, Italy; ^4^Parkinson's Disease and Movement Disorders Unit, I.R.C.C.S Hospital San Camillo, Via Alberoni 70, 30126 Venice, Italy; ^5^Nuclear Medicine Unit, San Raffaele Hospital, Via Olgettina 60, 20132 Milan, Italy; ^6^Vita-Salute San Raffaele University, Via Olgettina 58, 20132 Milan, Italy

## Abstract

This fMRI study deals with the neural correlates of spatial and objects working memory (SWM and OWM) in elderly subjects (ESs) and idiopathic Parkinson's disease (IPD). Normal aging and IPD can be associated with a WM decline. In IPD population, some studies reported similar SWM and OWM deficits; others reported a greater SWM than OWM impairment. In the present fMRI research, we investigated whether compensated IPD patients and elderly subjects with comparable performance during the execution of SWM and OWM tasks would present differences in WM-related brain activations. We found that the two groups recruited a prevalent left frontoparietal network when performing the SWM task and a bilateral network during OWM task execution. More specifically, the ESs showed bilateral frontal and subcortical activations in SWM, at difference with the IPD patients who showed a strict left lateralized network, consistent with frontostriatal degeneration in IPD. The overall brain activation in the IPD group was more extended as number of voxels with respect to ESs, suggesting underlying compensatory mechanisms. In conclusion, notwithstanding comparable WM performance, the two groups showed consistencies and differences in the WM activated networks. The latter underline the compensatory processes of normal typical and pathological aging.

## 1. Introduction

It has been reported that a consistent percentage of IPD patients (between 20–57% of the patients within the first 3–5 years after diagnosis) can evolve in Parkinson's disease mild cognitive impairment, with deficits in a single cognitive domain (i.e., amnestic or nonamnestic) or multiple domains. Compared with IPD patients without cognitive impairments, IPD-MCI subjects present up to 60% developing dementia (PDD) over a period of 2–5 years [1].

The pattern of cognitive impairments in IPD patients is often the one observed in patients with lesions in prefrontal cortex (PFC) with executive dysfunctions such as deficits in planning ability, problem solving, maintaining and shifting attention, behavioural regulation [2–7], and working memory [8–12]. The study of the frontal-like impairment in IPD patients has been largely focused on working memory. In particular, older studies have indicated that IPD patients could present a greater impairment in spatial working memory (SWM) compared to that in object working memory (OWM) tasks [13–20]. This pattern of impairments may reflect a predominant deficit of SWM in IPD [21], as well as a greater demand of executive requirements during spatial compared to nonspatial WM performances [20]. From a physiological point of view, it may be due to a greater disruption of circuits implicated in spatial processing [22] or a selective rescue of OWM network by dopaminergic medication [23]. On the contrary, more recent studies did not find this kind of dissociation [22–25].

A decline of working memory and executive functions (EF) was reported also in older age [26–28], and it has been related to changes in PFC functional activity. In a PET and fMRI meta-analysis by Rajah and D'Esposito [29] in elderly people, the PFC dysfunction was interpreted as a reduced regional process specificity and increases in nonspecific cortical activation. A research investigating aging-related functional alterations during the performance of a SWM task found bilateral activations in PFC in elderly subjects, in comparison to young volunteers that were instead confined to the left hemisphere. This bilateralization of activation agreed with the hemispheric asymmetry reduction in older (HAROLD) model, and it has been attributed to reduced capacities in elderly individuals to retain information in WM during the task execution, leading to a switch from proactive (seen in young adults) to reactive control strategies [28].

The visual system is divided into a ventral pathway, extending from the inferior temporal cortex to the ventrolateral prefrontal cortex (VLPFC), responsible for object identification, and a dorsal stream, extending from posterior parietal cortex (PPC) to dorsolateral prefrontal cortex (DLPFC), responsible for spatial location of objects [29]. According to this view, the SWM is mediated by a dorsal frontoparietal network, whereas the OWM is mediated by ventral temporal and frontal regions [30]. Consistently, Owen et al. in a meta-analysis about the n-back WM tasks in fMRI showed evidence for involvement of the left PFC in verbal WM, dorsal premotor in SWM, and right PFC in OWM [31]. Other studies, however, provided ambiguous results on the distinction of the frontal regions in spatial and non-SWM tasks. For example, a meta-analysis of 60 fMRI and PET studies found that the PFC is not selective for content type (spatial versus nonspatial) with the only exception of right PFC specialization for OWM. Consistent differences in material type were found to be limited to the posterior part of the brain, with a clear spatial versus object distinction [32].

On the distinction between the maintenance and the executive components of WM, several studies showed that the executive process of WM is amodal [33, 34], whereas the maintenance and manipulating process is likely organized by type of content (spatial versus nonspatial) [35, 36]. On the other hand, a recent meta-analysis from Nee and coworkers highlighted that the ventral and dorsal streams are both engaged in maintenance and executive processes of WM, and the region sensitive to nonspatial content is the midlateral prefrontal cortex, whereas the region sensitive to spatial content is located more dorsally in the caudal superior frontal sulcus [37].

Another meta-analysis including 189 fMRI experiments in healthy subjects during various working memory tasks showed a recruitment of the bilateral inferior frontal gyrus, left cerebellar lobule, and left ventral visual cortex in OWM, whereas, in SWM, there was a bilateral involvement of the posterior superior frontal gyrus, the superior parietal lobule, the precuneus, and the right inferior parietal cortex [38].

Thus, the existence of at least partially segregated neural networks for SWM and OWM has been considered in young age.

The goal of the present fMRI study was twofold. First, we wanted to examine possible changes in functional neural substrates in IPD in comparison to elderly normal controls during visual working memory tasks addressing spatial and object processing. Second, we wanted to explore the neural networks associated to object and spatial working memory task in aged healthy participants.

## 2. Materials and Methods

### 2.1. Subjects

Thirteen patients in the early stage of idiopathic Parkinson's disease (Hoehn and Yahr Stages I and II) [39] and twelve age-matched healthy volunteers participated in the study. The IPD patients were all outpatients at the Center for Movement Disorders, set in Milan, Italy. Patients with a diagnosis of IPD, in the absence of clinical dementia or depression symptoms, were included. The severity of clinical symptoms was assessed by the neurologist according to Hoehn and Yahr 5-point rating scale [39]. Only patients with a score between 1 and 2 were included in the present study. All patients were taking dopamine agonists and were tested in their “on” phase. Exclusion criteria for medicated patients included a significant medical history not directly related to IPD (i.e., stroke, psychiatric disorders, and head injury), mini mental state examination (MMSE) score below 24/30 [40], and Geriatric Depression Scale (GDS) [41] score above 10. Control volunteers (ESs) were recruited to be matched to the IPD group as closely as possible with respect to age and education. Exclusion criteria for the ESs group included any history of neurological or psychiatric diseases, substance abuse, or head injury. Clinical and demographic data of the two samples are reported in Table 1.

All participants and their caregivers gave informed consent to the experimental procedure, which was approved by the local Ethical Committee.

### 2.2. Cognitive Evaluation

Neuropsychological tests were performed in order to evaluate general cognitive profile and specific cognitive abilities, to make sure that none of the two groups were significantly impaired.

General cognitive abilities were evaluated in both groups using a series of standardized neuropsychological tests including mini mental state examination (MMSE) [40], digit span test forward and backward [42], and Corsi Block Test [43] for the evaluation of verbal and spatial short-term memory. Divided attention and set shifting abilities were assessed using Trail Making Test (parts A and B) [44]; the Letter Fluency Test [45] was used to assess lexical retrieval. We also assessed visuospatial long-term memory using the Visuospatial Supraspan Learning Test [46], and we used the Geriatric Depression Scale (GDS) [41] to be sure that performances, especially reaction times, were not compromised of depression.

### 2.3. Experimental Tasks

**Figure 1:**
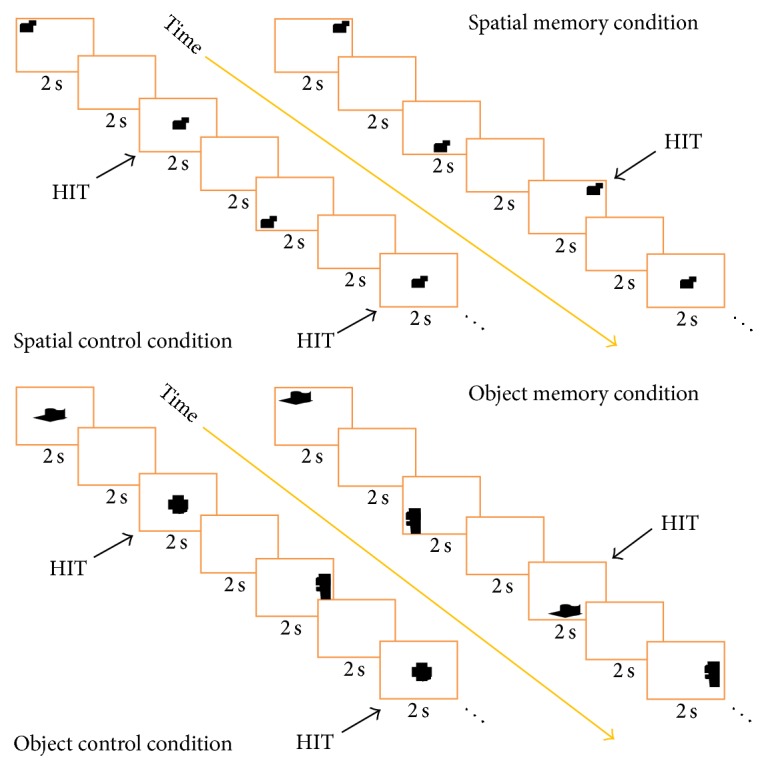
In the task condition, subjects had to press a key whenever they saw a repeated stimulus after 1 intervening stimulus. In the Spatial Memory Condition, they had to pay attention to the position of stimuli; in the Object Memory Condition, they had to focus on the shape of the stimuli.

We used a version of the 2-back working memory task [47] modified to distinguish object working memory from spatial working memory [48] (Figure 1). The 2-back is considered to be a working memory task because it requires temporary storage of the material and manipulation of the information to guide behavior [49, 50].

The participants were instructed to watch/look at a sequence of 9 stimuli presented one at a time and press a key whenever they saw a repeated stimulus after 1 intervening stimulus.

In the spatial working memory task (SWMT), stimuli appeared on the computer screen serially and pseudorandom in one of nine spatial locations. Participants pressed a key when a spatial location of a stimulus was repeated with one different intervening stimulus. The spatial working memory baseline (SWMB) was a simple detection task in which participants watched a succession of stimuli appearing at different locations on the screen and responded each time a stimulus appearing in the center position.

In the object working memory task (OWMT), stimuli of different shapes appeared in one of the nine different spatial locations described above in pseudorandom order. Participants pressed a key when a stimulus with a certain shape was repeated with one different intervening stimulus. In the object working memory baseline (OWMB), participants watched a succession of stimuli with different shapes and responded each time a prelearned target appeared. Stimuli for both spatial and object tasks were abstract targets, so they were distinct but difficult to verbalize [51].

Each subject performed a total of 4 blocks, two for the spatial and two for the object condition. Half subjects were presented with the spatial condition first and the other half with the object condition first. In each block, there was a fixed alternation of task and baseline according to the sequence task/baseline/task/baseline. An 8 seconds instruction message appeared before each block followed by 18 stimuli appearing for 2 seconds and an ISI of 2 seconds. Each block was 80 seconds long. The total time of the experiment was about 21 minutes.

Performance in behavioral tasks was evaluated considering both reaction times and errors. Both false positive (pressing the key when it was not the right answer) and false negative (not pressing the key when it was the right answer) were calculated as errors.

### 2.4. fMRI Methods

#### 2.4.1. Acquisition

Brain imaging was conducted on a 1.5 Tesla Sigma scanner (General Electric, Milwaukee, WI, USA) equipped with a standard head coil. Twenty-four contiguous, gradient-echo echo planar images, sensitive to BOLD contrast, parallel to the AC-PC, were acquired using a T2^*^ weighted gradient-echo EPI sequence (TR 4 sec, TE 4 sec, FOV 28 cm, image matrix 64^2^, flip angle 90°, slide thickness 4 mm, and cubic 4.375 mm^3^ isotropic voxels). High-resolution T1-weighted images were also acquired (TR 4.50 sec, TE 9 msec, FOV 24 cm, matrix 256^2^, and slice thickness 1.5 mm).

#### 2.4.2. Images Processing

Data preprocessing and statistical analyses were performed using Statistical Parametric Mapping SPM2 software (Wellcome Department of Cognitive Neurology, London, UK) implemented in Matlab (Mathworks Inc., Sherborn, Ma, USA). Functional images were corrected for acquisition order and realigned to the first functional images for each participant; motion artifacts were corrected with SPM-2 procedures by using realignment parameters. Anatomical and functional images were spatially normalized to stereotactic space in the Montreal Neurological Institute (MNI305) template using 7 × 8 × 7 nonlinear functions. The functional images were resampled into 2 × 2 × 4 volume voxels and then smoothed using an 8 mm isotropic Gaussian kernel.

#### 2.4.3. Data Analysis

Statistical analyses were performed separately for SWM and OWM. For each participant, task-related activity was identified using the general linear model as implemented in SPM-2 to model the effects of interest and the confounding effects (age, session effects, and magnetic field drift). Two types of group analyses were conducted using random-effects models [52, 53]. First, the two conditions (SWMT and OWMT) were compared to their baselines, and a conjunction of the two activation maps for each group was calculated using the ImCalc feature in SPM 2 [54]. Second, the two groups of participants were compared directly for each experimental condition: SWMT, baseline in IPD, versus SWMT, baseline in ESs; OWMT, baseline in IPD versus OWMT, baseline in ESs, and vice versa.

Significance threshold was set at *P* < 0.001, uncorrected in all comparisons; in the between-group comparisons, it was set at *P* < 0.005, uncorrected. Only activations with more than 10 voxels were considered.

In order to investigate activations in basal ganglia and thalamus, we performed a ROI analysis using a SPM-2 toolbox, the MarsBar toolbox, which can compute comparisons (i.e., SWMT, baseline) on specific ROIs (MarsBar program: http://marsbar.sourceforge.net/). We therefore obtained a contrast value for each subject in each investigated region, namely, caudate, putamen pallidum, and thalamus. Than we performed a Mann-Whitney *U* test statistic in order to evaluate differences between groups (the choice of a nonparametric statistic test is due to the low number of data we had to compare).

We were also interested in understanding which brain areas were correlated to how good the way the task was performed: we therefore created a “performance score” for each subject applying a transformation to the data using the formula 100 − [(subject value − lowest value of all subjects/highest value) ∗ 100], in which the subject values represent the result of the multiplication of reaction 8 time by number of mistakes. In this way, we considered both accuracy and speed in one value on a range between 0 and 100. Afterwards, using a random effect in SPM2 for both SWMT and OWMT, we performed a correlation analysis with the “performance score” and the brain activity. We obtained a positive correlation (more brain activity as the subject performs better during the task) and a negative correlation (more brain activity as the subject performs worse during the task). We also performed a correlation analysis based on regions of interest in the basal ganglia and the performance score.

## 3. Results

### 3.1. Behavioral Results

#### 3.1.1. Cognitive Evaluation

IPD patients reported normal scores in all the tests used to evaluate general cognitive abilities. Statistical analysis (paired *t*-test) showed no significant differences in the cognitive performance between IPD and ESs (see Table 1).

#### 3.1.2. Experimental Tasks

All subjects, IPD and ESs, performed the WM tasks above the threshold of 50% correct (mean percent calculated after applying correction for false positive and false negative responses). Paired *t*-test revealed no significant differences between the two groups neither in the performance scores nor in the reaction times (Table 2).

Mean scores were a bit lower in OWM task than in SWM in both groups, but it was not significant. It is possible that this task became harder for elderly people.

### 3.2. fMRI Results

#### 3.2.1. Within-Group Comparisons

**Figure 2:**
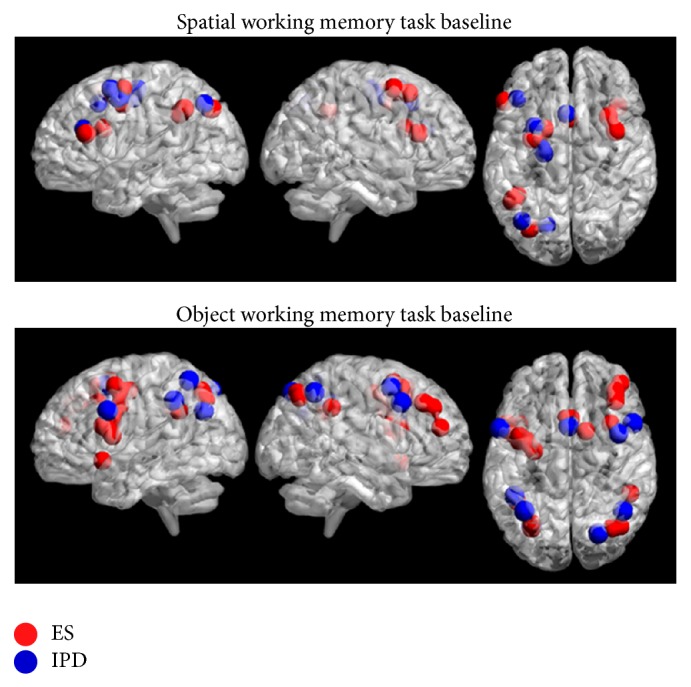
Image shows SPM within-group comparisons for the SWMT-SWMB and OWMT-OWMB contrasts. 3D reconstruction of SPM contrasts (BrainNet viewer [69]). Spherical ROIs depicting peaks of activation in Cluster extent of *k* = 10 voxels; *P* value < 0.001, uncorrected in all comparisons (see text for details on the stereotactic coordinates).

For spatial working memory versus baseline, IPD patients and ESs showed a similar pattern of activation. Both groups recruited a left frontoparietal network including lateral prefrontal cortex (BA46), superior frontal gyrus (BAs 8 and 6), premotor and supplementary motor cortex (BA6) and parietal areas (BAs 7 and 40). In IPD patients, the voxel extension of the activated left sided network was significantly larger (on a simple *t*-test) compared to that in ESs (in IPD total 349 voxel, in ESs total 214 voxel). ESs showed additional activations in the right hemisphere in lateral prefrontal cortex (BA46) and superior frontal gyrus (BAs 8 and 6), while in IPD the right hemispheric activation was not present (Figure 2).

For a view of the IPD and ESs brain activation in SWMT and SWMB, see Table 3.

For object working memory versus baseline, in OWMT both groups recruited left and right hemispheric structures. In particular, ESs showed bilateral activations in the superior frontal gyrus (BA8), premotor cortex (BA6), and inferior parietal lobe (BA40); left sided activations were localized in the inferior frontal gyrus (BAs 44 and 6), superior parietal lobe (BA7), and temporal pole (BA38) whereas the right sided activation was in the dorsolateral prefrontal cortex (BAs 9 and 8). The IPD patients showed bilateral activations in the inferior frontal gyrus (BAs 14, 44, and 6), more extended on the left side; left sided activation in the premotor cortex (BA6) and inferior parietal lobe (BA40); right sided activations in the superior frontal gyrus (BA8) and superior parietal lobe (BA7). The precuneus (BA7) was activated bilaterally in ESs and IPD group (see Figure 2).

For a view of the IPD and ESs brain activation in OWMT and OWMB, see Table 4.

#### 3.2.2. Between-Group Comparison

In ESs, the between-group comparison for spatial working memory versus baseline showed activations in the left superior frontal gyrus (BA8 −26 0 44) and right superior parietal cortex (BA5/7 22 −32 60). IPD patients showed activations in left inferior parietal cortex (BA40 −28 −54 48) and left middle temporal gyrus (BA21 −62 −28 0).

In ESs, the between-groups comparison for object working memory versus baseline revealed left sided activations in the middle frontal gyrus (BA8 28 2 48), inferior parietal lobule (BA40 −44 −30 −52), temporal pole (BA38 −40 16 −5), and right-sided activations in superior frontal gyrus (BA8 −42 14 44) and premotor cortex (BA6 14 4 60). In the IPD group, no regions showed greater activation than those in the ESs group.

#### 3.2.3. ROIs Analysis

For the SWMT, we did not find any difference between IPD and ESs in the selected ROIs. On the other hand, ESs showed a greater neural activity than IPD bilaterally in caudate, pallidum, and putamen during the OWMT.

#### 3.2.4. Correlation Analysis with the Performance Score in IPD

In spatial working memory task, IPD subjects show a positive correlation between “performance score” and neural activity in the middle temporal region (BA21) bilaterally, whereas there is a negative correlation in the right precuneus (BA7) and the cingulate gyrus (BA24).

In object working memory task, IPD subjects show a positive correlation in the left inferior frontal gyrus (BAs 46, 47, 10, and 46) and bilateral superior frontal gyrus (BA8); brain areas involved in the negative correlation are precentral sulcus bilaterally (BA6) and the left thalamus.

ROIs analysis did not show any positive or negative correlation between “performance score” and activation in the basal ganglia.

## 4. Discussion

Overall, significant brain activation in the IPD group was more extended (voxel extent) than that in the ESs group, both in SWMT and OWMT. It is possible that the IPD patients used more variable and distributed neural substrates than ESs to perform comparably the WM tasks. Marié and coworkers found a similar result comparing IPD patients with control subjects during the executions of short and long-term WM tasks. IPD patients presented a greater activation than controls in right prefrontal (BA9) and posterior parietal (BAs 40 and 7) regions when the short-term WM task was performed. The authors considered this enhanced recruitment of frontoparietal network to be suggestive of patients' efficient compensatory mechanisms [55].

During OWM and SWM task, both groups activated the left superior frontal gyrus, left parietal foci, and left premotor cortex. These data indicate that there is an overlap of brain structures involved in SWM and OWM in the left hemisphere which is also consistent with the left prevalent activation in younger subjects [28].

The common left hemispheric laterality might be also explained by the fact that both groups were using a verbal strategy performing the two tasks. Moreover, participants might have endorsed a mental counting of the order of the sequence they have to keep in mind to produce a correct answer. The prefrontal and parietal cortices were already found during arithmetic tasks execution. In particular, left parietal cortex activation was associated with optimization of mathematical abilities, and left frontal cortex was associated with increase of calculation complexity, as well as linguistic and working memory functions [56].

In addition, the present fMRI study confirms the presence of partially segregated systems for SWM, that is, a dorsal frontoparietal network, and for OWM, that is, a frontotemporal network in the two groups. More specifically, both healthy aged adults and IPD patients recruited a prevalent left frontoparietal network during performance of SWM and more bilateral networks in OWM tasks (Figure 2).

For SWM, ESs and IPD patients rely also on visuospatial processes localized in posterior parietal cortex. Activations in inferior and superior parietal lobules for SWMT have been already reported in other studies [32]. The between-group comparison in SWM tasks shows that ESs recruit specifically the left superior frontal regions and the right superior parietal cortex, whereas IPD patients seem to rely more on left temporal and parietal areas. Taken together, these findings suggest that IPD patients may need different resources to perform the task, consistent with the previously documented deficit in SWM.

For OWM, ESs recruited a bilateral frontoparietal network, including the middle, superior, and inferior frontal gyri, premotor cortex, and superior and inferior parietal lobes. There were also selective activations in the left temporal pole, a region implicated in representation of visual objects [57], and in the bilateral precuneus found by Kaiser et al. (2010), activated during the use of mental imagery strategy in OWM tasks [58]. IPD showed smaller activation foci in a comparable bilateral neural system. In both groups, there was an activation of the left inferior frontal gyrus that might be attributed to a verbal component involved in the task, suggesting that subjects tried to name the shape despite being abstract. Some subjects indeed reported having used such a strategy. Furthermore, this result is in agreement with previous fMRI studies performed in healthy subject, reporting that the inferior prefrontal cortex is an important region distinguishing nonspatial versus spatial WMT [35, 59–61].

Along with the above mentioned activations, ESs and IPD patients showed SMA and pre-SMA foci. The pre-SMA is a region implicate in higher-order motor control [28]. Previous studies attributed to these regions a role in spatial rehearsal [62, 63] or motor imagery [64].

With ROIs analysis in subcortical gray structures, we demonstrated differences in basal ganglia activation during OWMT. ESs showed greater activation than IPD in caudate, putamen, and pallidus, bilaterally. Better activation in the basal ganglia regions in ESs than IPD is probably due to the underline pathology. We might have expected these functional changes also in the SWMT; however, since we choose IPD patients at initial disease stage and pharmacologically well compensated, differences may have been so small that we were not able to reveal them. Alternatively, we were able to see this different activation only with OWMT because the nigrostriatal network has been seen to be more involved in this kind of tasks.

Our correlation analysis with the performance scores showed that IPD patients with a better performance during the OWMT activated a frontal system, that is, bilateral superior frontal gyrus and left inferior frontal gyrus, overlapping to the one activated in ESs during the performance on the same task.

Regarding the normal ageing effects, during SWM task execution, ESs recruited a left frontoparietal network presenting additional activations in the right hemisphere, namely, in the middle frontal gyrus and superior frontal gyrus. Previous studies in elderly population showed a reduction of prefrontal lateralization with respect to young subjects during the performance of tasks requiring executive capacities [28]. This lack of lateralization has been considered as a model of hemispheric asymmetry reduction in older age (HAROLD) [54, 65, 66]. Our results support HAROLD model, given the presence of a bilateral activation in ESs group. Noteworthy, this effect was not found in IPD patients. They showed indeed a limited left-sided activation that however extended beyond the typical SWM network, thus suggesting the presence of different underlying compensatory processes.

In conclusion, our results indicate that the group of compensated IPD patients was able to perform the object and spatial WM tasks as well as the control group.

We found that, notwithstanding a conservation of OWM and SWM abilities, there were consistencies and differences in brain network activations. In particular, IPD patients showed a less compensatory capacity and a reduced frontostriatal network compared to ESs, probably due to the underlying pathology. The striatum projects ~80% of its total volume to the frontal cortex, and these projections are mainly subserving executive functions, representing a large portion of the corticostriatal loop [67, 68].

Our results confirmed that the SWM and OWM are mediated by distinct, even if not completely segregated, networks in aging. The bilateral activity in elderly subjects, during the execution of SWM task, suggests the presence of aging-related compensatory mechanisms (HAROLD model).

## Figures and Tables

**Table 1:** Clinical and demographic data of IPD patients and healthy volunteers (ESs) (mean ± SD).

	IPD (mean ± SD)	ESs (mean ± SD)	Statistics
Age	63.3 ± 6.3	59.0 ± 2.3	Ns
Education	13.4 ± 4.4	13.9 ± 7.2	Ns
Disease duration	5 ± 3.4		—
MMSE	29.27 ± 0.65	28.91 ± 1.04	Ns
DIGIT SPAN forward	6.55 ± 0.82	6.82 ± 0.75	Ns
Digit span backward	4.36 ± 0.81	5.09 ± 1.38	Ns
Corsi blocks	4.91 ± 0.94	5. 18 ± 1.8	Ns
Letter fluency	28.64 ± 8.19	37.64 ± 7.03	Ns
Trail making A (sec.)	49.36 ± 22.09	32.64 ± 6.67	Ns
Trail making B (sec.)	101. 82 ± 43.77	67.00 ± 17.79	Ns
Visuospatial supraspan	12.55 ± 4.27	10.27 ± 3.72	Ns
GDS	3.82 ± 2.52	2.82 ± 2.52	Ns

No statistical differences at *P* < 0.001.

**Table 2:** Correct responses (%) and RTs (ms) of behavioural performance score (mean ± SD).

	IPD patients		ESs	
	% Correct	RTs (ms)	% Correct	RTs (ms)
SWMT	81.07 ± 13.51	649 ± 101	91.09 ± 16.01	628.08 ± 156.05
SWMB	92.08 ± 11.15	690.09 ± 193.07	93.55 ± 0.64	654.09 ± 214.01
OWMT	68.55 ± 19.05	950.85 ± 170.07	74.07 ± 19.06	1159.01 ± 1007
OWMB	100	671.01 ± 122.01	98.03 ± 5.03	700 ± 216.75

Statistical analysis found no significant differences between the two groups.

**Table 3:** Within-groups comparisons SWMT baseline.

BA	Anatomical area	ESs										IPD									
		Left hemisphere					Right hemisphere					Left hemisphere					Right hemisphere				
		*X*	*Y*	*Z*	*Z*-sc	*K*	*X*	*Y*	*Z*	*Z*-sc	*K*	*X*	*Y*	*Z*	*Z*-sc	*K*	*X*	*Y*	*Z*	*Z*-sc	*K*
46	Middle frontal gyrus	−52	28	20	3.95	25	40	24	20	4.12	14	−42	30	24	4.33	14					
		−44	32	24																	
8.6	Sup. frontal gyrus	−30	−2	56	4.97	55	32	16	52	3.72	25	−30	2	48	4.13	82					
		−24	−2	44	3.87		38	4	56	3.18		−28	8	56	3.77						
		−20	6	52	3.28							−40	0	48	3.92						
												−36	−6	52	3.17						
6	Pre SMA	0	14	24	3.77	26						−2	18	44	3.96	23					
6	SMA											−22	−10	48	3.43	15					
												−20	−14	56	3.19						
7	Sup. parietal lobule	−32	−74	40	3.96	108						−26	−72	52	4.38	215					
40	Inf. parietal lobule	−46	−52	40								−38	−68	44	4.17						
		−42	−48	36																	
7	Precuneus											−18	−72	40	4.03						

Activated areas by ESs group (left) and IPD group (right) during the performance of SWMT. BA: Brodmann area; Sup.: superior; Inf.: inferior; *Z*-sc: *Z*-score, *P* < 0.005; *K*: number of voxels in each cluster. (See also Figure 2.)

**Table 4:** Within-groups comparisons in OWMT baseline.

BA	Anatomical area	ESs										IPD									
		Left hemisphere					Right hemisphere					Left hemisphere					Right hemisphere				
		*X*	*Y*	*Z*	*Z*-sc	*K*	*X*	*Y*	*Z*	*Z*-sc	*K*	*X*	*Y*	*Z*	*Z*-sc	*K*	*X*	*Y*	*Z*	*Z*-sc	*K*
8	Sup. frontal gyrus	−40	4	52	3.82	95	34	14	52	4.11	1 76						40	6	52	3.75	13
		−28	−8	48	3.62																
		−34	0	48	3.49																
44/6	Inf. frontal gyrus	−46	6	24	4.44	95						−56	10	32	3.50	10	52	14	40	4.72	10
		−40	0	36	3.65																
		−50	6	12	3.54																
6	Pre-SMA	−2	18	52	3.69		2	18	44	4.42	95	0	12	52	3.32	11					
							14	8	56	4.05											
9/8	Mid. frontal gyrus						42	46	20	4.57	47										
							38	40	36	3.74											
							38	32	40	3.38											
7	Sup. parietal lobule	−30	−66	48	4.83	324											44	−56	48	4.51	262
40	Inf. parietal lobule	−42	−48	32	4.52		50	−42	36	3.89	163	−44	−44	40	4.53	45					
7	Precuneus	−30	−70	40	4.10		42	−68	44	3.72		−36	−56	56	3.83		24	−74	48	4.39	16
							34	−70	48	3.82		−30	−68	32	3.72		12	−78	40	3.64	
38	Temporal pole	−50	14	−20	4.00	45															
		−40	16	−8	3.85																

Activated areas by ESs group (left) and IPD group (right) during the performance of OWMT. BA: Brodmann area; Sup.: superior; Inf.: inferior; Mid.: middle; *Z*-sc: *Z*-score, *P* < 0.005; *K*: number of voxels in each cluster. (See also Figure 2.)

## Abbreviation List

IPD: Idiopathic Parkinson's disease
MCI: Mild cognitive impairment
SWMT: Spatial working memory task
SWMB: Spatial working memory baseline
OWMB: Object working memory baseline
OWMT: Object working memory task
ES: Elderly subject
fMRI: Functional magnetic resonance
MMSE: Mini mental state examination
PFC: Prefrontal cortex
VLPFC: Ventrolateral prefrontal cortex
PPC: Posterior parietal cortex
DLPFC: Dorsolateral prefrontal cortex
EF: Executive functions
GDS: Geriatric depression scale
ROI: Region of interest
HAROLD: Hemispheric asymmetry reduction in older
BA: Brodmann area.
